# Supplementary material for: pH and Reduction Dual-Responsive Bi-Drugs Conjugated Dextran Assemblies for Combination Chemotherapy and In Vitro Evaluation
Source: Polymers (Basel). 2021 May 8;13(9):1515. doi: 10.3390/polym13091515 (PMC8125917; doi:10.3390/polym13091515)
Supplement: Supplementary file 1 [file polymers-13-01515-s001.zip › polymers-1172944-supplementary.pdf]

Supporting Information

# pH and Reduction Dual-Responsive Bi-Drugs Conjugated Dextran Assemblies for Combination Chemotherapy and In Vitro Evaluation

**Xiukun Xue [1], Yanjuan Wu [1,*], Xiao Xu [2], Ben Xu [1], Zhaowei Chen [2,*] and Tianduo Li [1,*]**

1 Shandong Provincial Key Laboratory of Molecular Engineering, School of Chemistry and Chemical Engineering, Qilu University of Technology (Shandong Academy of Science), Jinan 250353, China; Jasonxue@yeah.net (X.X.); xuben2019@126.com (B.X.)

2 Institute of Food Safety and Environment Monitoring, College of Chemistry, Fuzhou University, Fuzhou 350108, China; 201310023@fzu.edu.cn

* Correspondence: wuyanjuan5@qlu.edu.cn (Y.W.); chenzw@fzu.edu.cn (Z.C.); ylpt6296@vip.163.com (T.L.)

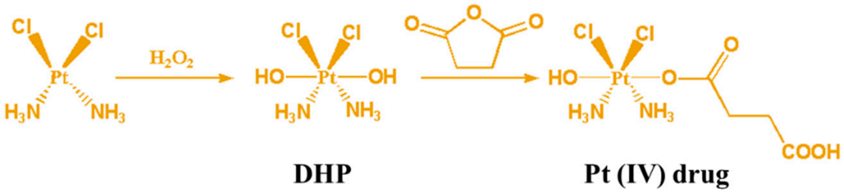


**Scheme S1.** Synthetic scheme of the DHP (diamminedichloro-dihydroxyplatinum) and Pt (IV) prodrug (succinic anhydride modified DHP).

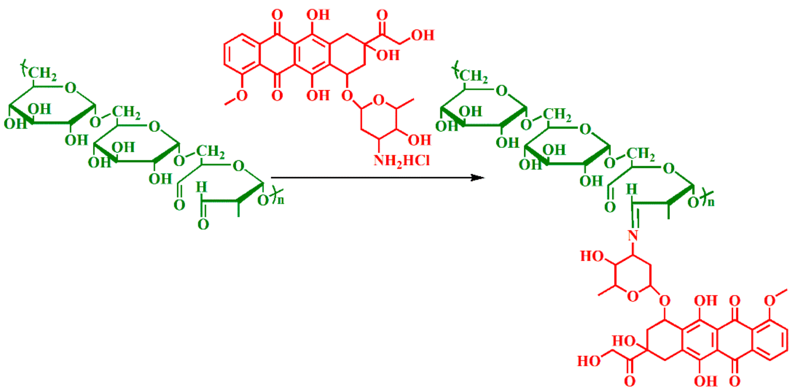

**Scheme S2.** Synthetic scheme of the oDex-g-DOX (DOX conjugated oxidized dextran).

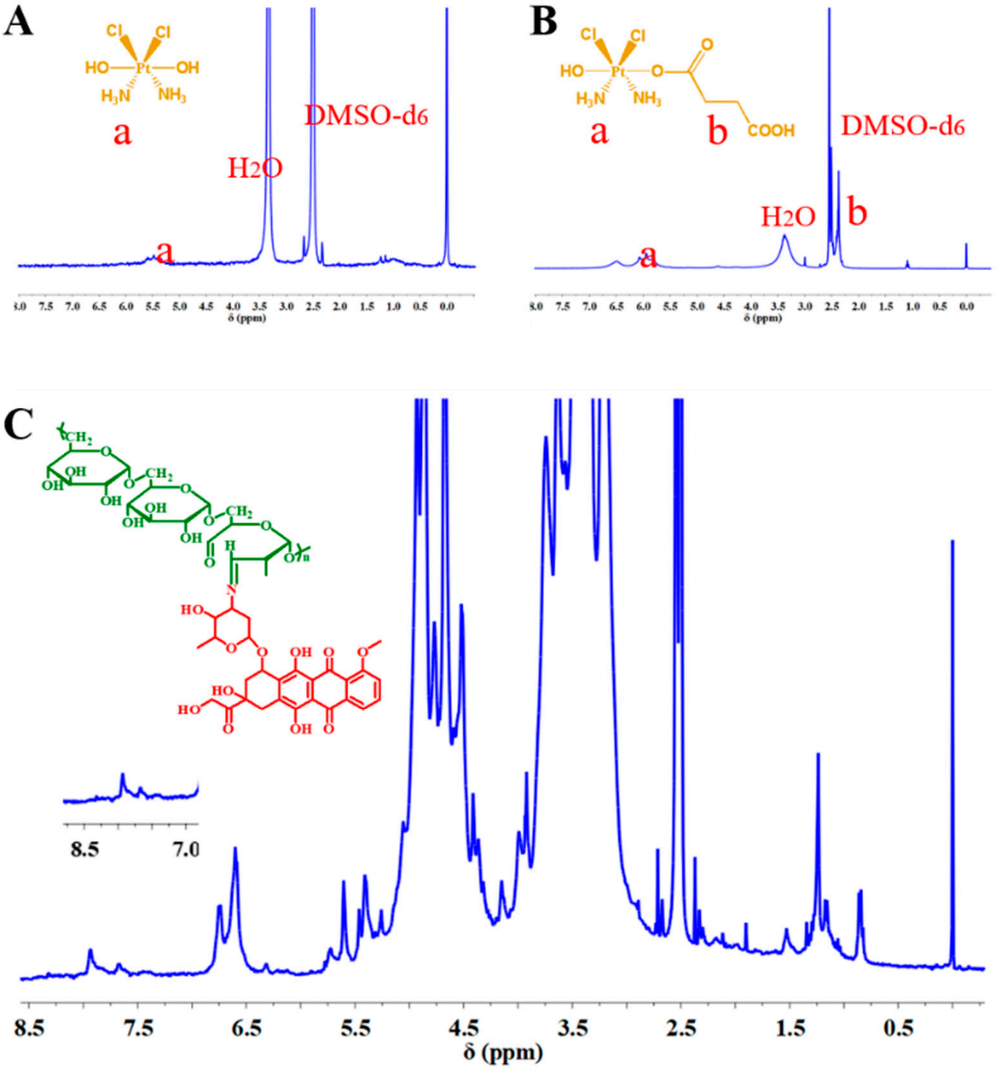


**Figure S1.** The $^{1}$H NMR spectra of DHP (diamminedichloro-dihydroxyplatinum) (**A**), Pt (IV) prodrug (succinic anhydride modified DHP) (**B**), and oDex-g-DOX (DOX conjugated oxidized dextran) (**C**) in DMSO-$d_6$.

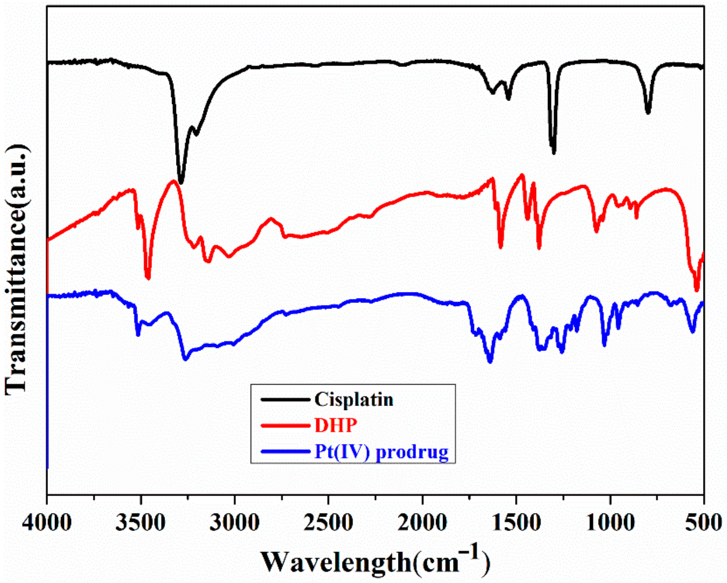


**Figure S2.** Fourier-transform infrared spectra (FT-IR) spectra of the cisplatin, DHP (diamminedichloro-dihydroxyplatinum) and Pt (IV) prodrug (succinic anhydride modified DHP).

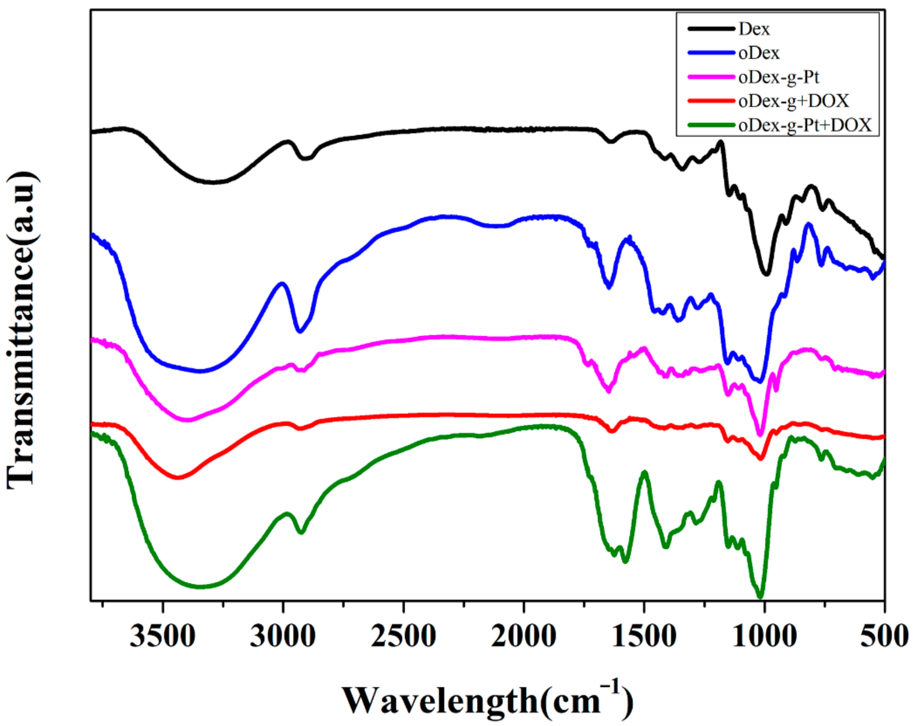


**Figure S3.** Fourier-transform infrared spectra (FT-IR) spectra of the Dex (dextran), oDex (oxidized dextran), oDex-g-Pt (platinum conjugated oxidized dextran), oDex-g-DOX (DOX conjugated oxidized dextran) and oDex-g-Pt+DOX (platinum plus DOX conjugated oxidized dextran).

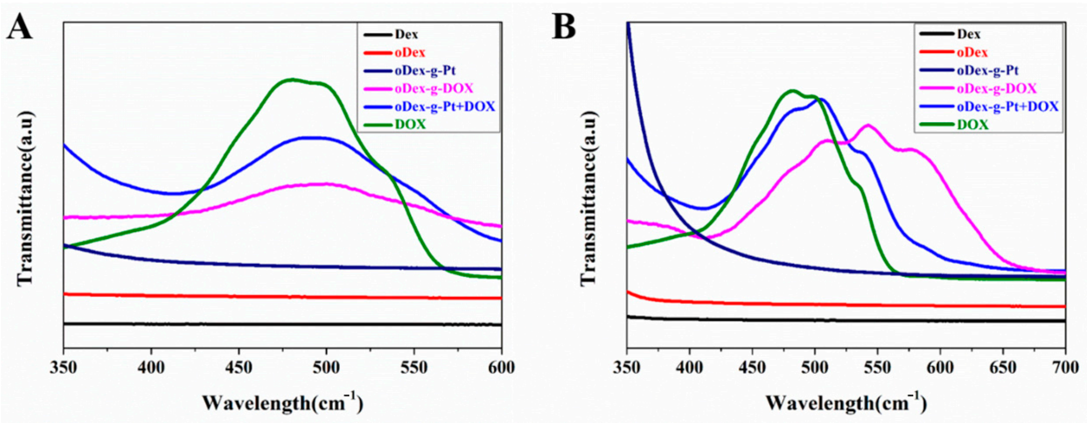


**Figure S4.** UV–visible spectrum of DOX, Dex (dextran), oDex (oxidized dextran), oDex-g-Pt (platinum conjugated oxidized dextran), oDex-g-DOX (DOX conjugated oxidized dextran) and oDex-g-Pt+DOX (platinum plus DOX conjugated oxidized dextran) in aqueous solution (**A**) and DMSO (**B**).

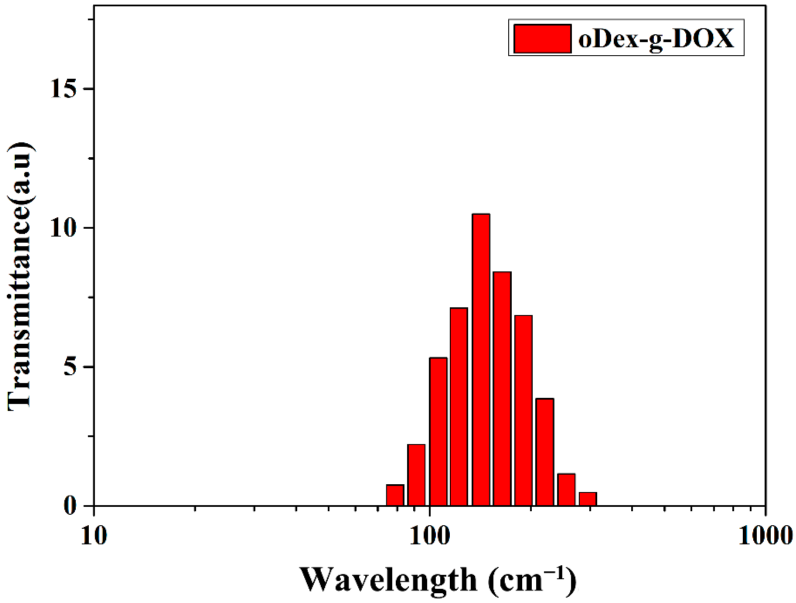


**Figure S5.** Size and size distribution of oDex-g-DOX NPs (DOX conjugated oxidized dextran nanoparticles).

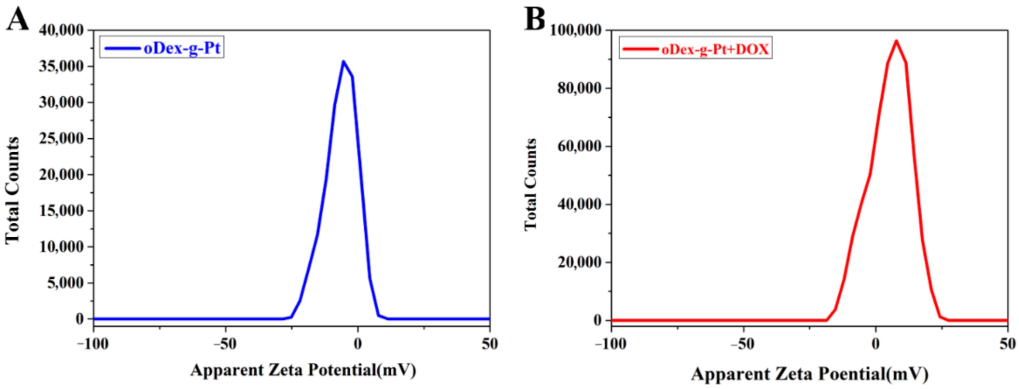


**Figure S6.** Zeta potential measurement of oDex-g-Pt (platinum conjugated oxidized dextran) (A) and oDex-g-Pt+DOX (platinum plus DOX conjugated oxidized dextran) (B).

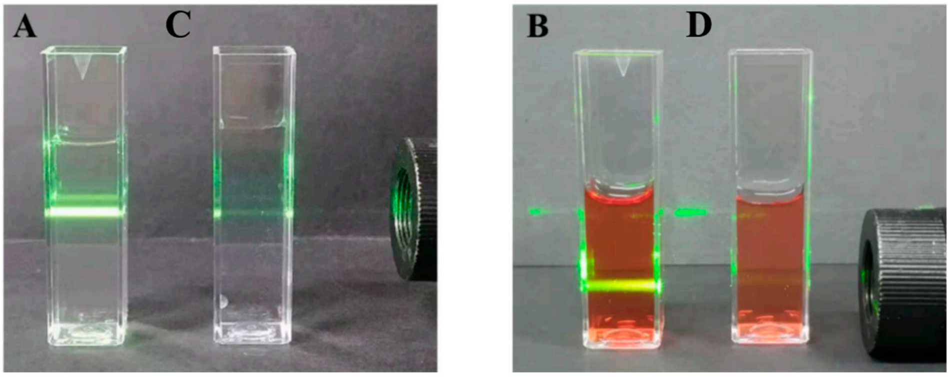


**Figure S7.** Tyndall effect seen for oDex-g-Pt NPs (platinum conjugated oxidized dextran nanoparticles) (**A**), oDex-g-Pt NPs cultured in pH 7.4 with 10 mM GSH for 24 h (**B**), oDex-g-Pt+DOX NPs (platinum plus DOX conjugated oxidized dextran nanoparticles) (**C**), and oDex-g-Pt+DOX NPs cultured in pH 5.0 with 10 mM GSH for 24 h (**D**).

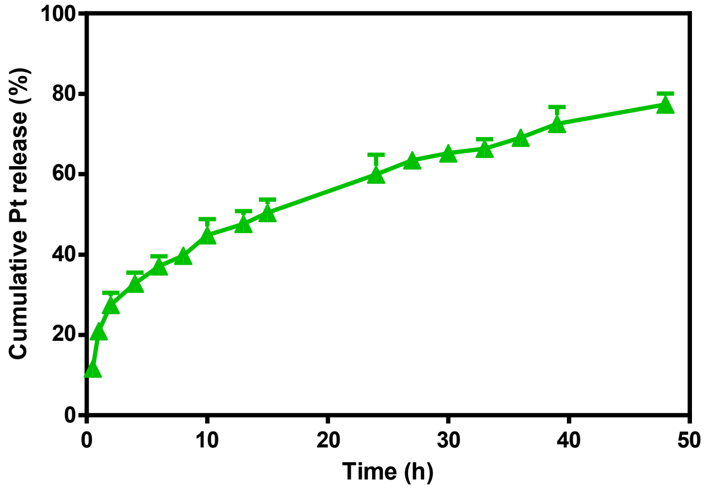


**Figure S8.** Drugs release profiles. Pt release behaviors from oDex-g-Pt+DOX NPs (platinum plus DOX conjugated oxidized dextran nanoparticles) in the presence of 20 mM GSH.

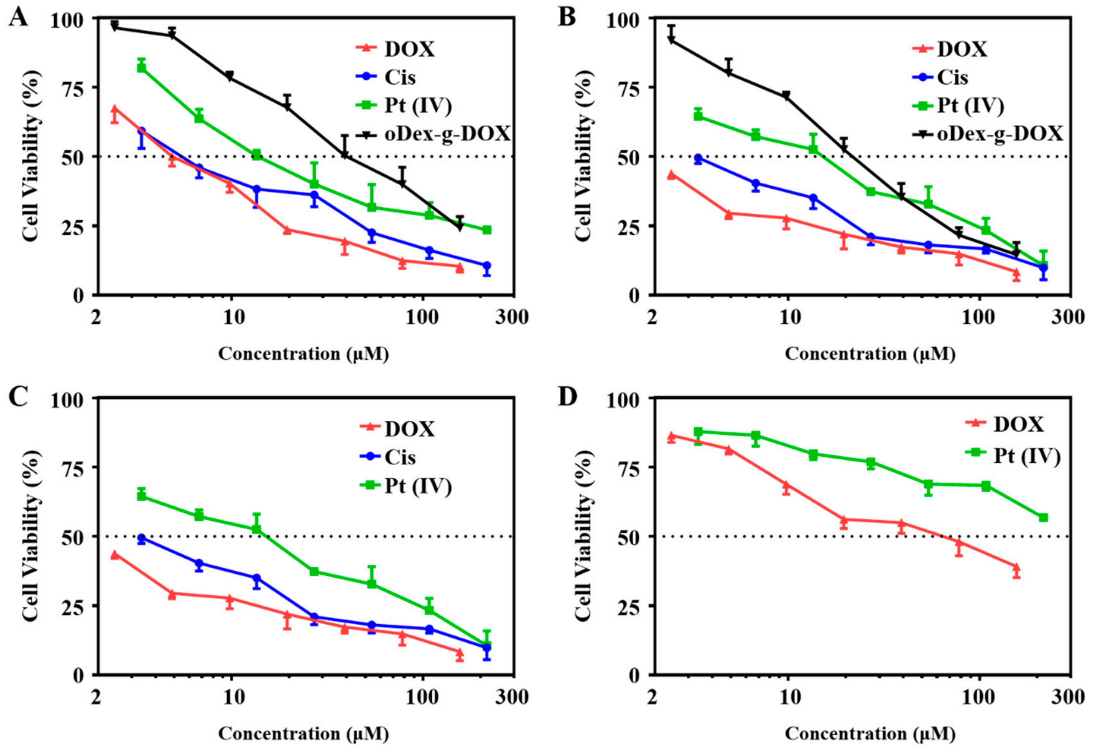


**Figure S9.** *In vitro* anti-cancer efficacy of drugs and nanodrugs. Cell viability curves of HeLa cells incubated with drugs and nanodrugs for 48 h (**A**) or 72 h (**B**). Cell viability curves of A549 cells (**C**) and A549/DDP cells (**D**) after incubation with drugs and nanodrugs for 48 h.
